# Use of real-time multiplex PCR, malaria rapid diagnostic test and microscopy to investigate the prevalence of *Plasmodium* species among febrile hospital patients in Sierra Leone

**DOI:** 10.1186/s12936-020-03163-2

**Published:** 2020-02-21

**Authors:** Tomasz A. Leski, Chris Rowe Taitt, Abdulai G. Swaray, Umaru Bangura, Nathanael D. Reynolds, Andrew Holtz, Chadwick Yasuda, Joseph Lahai, Joseph M. Lamin, Victoria Baio, Kathryn H. Jacobsen, Rashid Ansumana, David A. Stenger

**Affiliations:** 1 Center for Bio/Molecular Science & Engineering, U.S. Naval Research Laboratory, Washington, DC USA; 2 College of Science, George Mason University, Fairfax, VA USA; 3 Mercy Hospital Research Laboratory, Bo, Sierra Leone; 4 Department of Global & Community Health, George Mason University, Fairfax, VA USA; 5 Njala University, Bo Campus, Bo, Sierra Leone

**Keywords:** Malaria, *Plasmodium*, *Plasmodium falciparum*, *Plasmodium malariae*, *Plasmodium ovale*, Multiplex polymerase chain reaction, Immunoassay, Microscopy, Sierra Leone

## Abstract

**Background:**

Malaria continues to affect over 200 million individuals every year, especially children in Africa. Rapid and sensitive detection and identification of *Plasmodium* parasites is crucial for treating patients and monitoring of control efforts. Compared to traditional diagnostic methods such as microscopy and rapid diagnostic tests (RDTs), DNA based methods, such as polymerase chain reaction (PCR) offer significantly higher sensitivity, definitive discrimination of *Plasmodium* species, and detection of mixed infections. While PCR is not currently optimized for routine diagnostics, its role in epidemiological studies is increasing as the world moves closer toward regional and eventually global malaria elimination. This study demonstrates the field use of a novel, ambient temperature-stabilized, multiplexed PCR assay in a small hospital setting in Sierra Leone.

**Methods:**

Blood samples from 534 febrile individuals reporting to a hospital in Bo, Sierra Leone, were tested using three methods: a commercial RDT, microscopy, and a Multiplex Malaria Sample Ready (MMSR) PCR designed to detect a universal malaria marker and species-specific markers for *Plasmodium falciparum* and *Plasmodium vivax*. A separate PCR assay was used to identify species of *Plasmodium* in samples in which MMSR detected malaria, but was unable to identify the species.

**Results:**

MMSR detected the presence of any malaria marker in 50.2% of all tested samples with *P. falciparum* identified in 48.7% of the samples. *Plasmodium vivax* was not detected. Testing of MMSR *P. falciparum*-negative/universal malaria-positive specimens with a panel of species-specific PCRs revealed the presence of *Plasmodium malariae* (n = 2) and *Plasmodium ovale* (n = 2). The commercial RDT detected *P. falciparum* in 24.6% of all samples while microscopy was able to detect malaria in 12.8% of tested specimens.

**Conclusions:**

Wider application of PCR for detection of malaria parasites may help to fill gaps existing as a result of use of microscopy and RDTs. Due to its high sensitivity and specificity, species coverage, room temperature stability and relative low complexity, the MMSR assay may be useful for detection of malaria and epidemiological studies especially in low-resource settings.

## Background

Of the more than 200 million malaria cases estimated to occur each year, the overwhelming majority (90%) take place in sub-Saharan Africa [1]. Sierra Leone in particular has one of the highest burdens of malaria, with an estimated 3 million cases and 17,600 deaths occurring in 2017 [1]. Rapid detection of the causative pathogens is an essential component of effective management of malaria. *Plasmodium falciparum* is responsible for 99% of malaria infections in Africa, while *P. vivax* causes the majority of infections in other parts of the world*. Plasmodium knowlesi*, which until recently was believed to infect only monkeys, was found a common cause of human infections in Southeast Asia [2]. *Plasmodium ovale* and *Plasmodium malariae* are less commonly diagnosed species of malaria parasite found mostly in Africa but are also detected elsewhere [3, 4]. *Plasmodium vivax* and *P. ovale* require use of specialized anti-malarials to eliminate dormant stages that can cause relapses [5]. It is therefore important for the malaria species to be determined as part of diagnosis.

Giemsa-stained thick and thin film microscopy is still considered the gold standard in malaria diagnostics, as it is relatively sensitive, quantitative, and allows for species identification. Since many resource-poor environments lack widespread expertise in microscopy and high quality equipment, the World Health Organization (WHO) recommends the use of rapid diagnostic tests (RDTs) in these settings [6]. RDTs are less sensitive than microscopy but they are considerably easier to perform and do not require additional equipment. The utility of commercial RDTs is somewhat limited, however, because they are usually optimized for detection of *P. falciparum* and/or *P. vivax*, but not *P. malariae* or *P. ovale*, and sensitivity for the latter species is significantly lower [1, 7–9]. As a result, the prevalence of *P. malariae* and *P. ovale* (and potentially *P. vivax*) may be significantly underestimated [10]. Another recently discovered weakness of RDTs is their failure to detect strains of *P. falciparum* with deletion of histidine-rich protein 2 and 3, which are relatively common in certain regions [11, 12].

DNA-based diagnostics, also referred to as Nucleic Acid Amplification Tests (NAATs), such as DNA/RNA hybridization, conventional and real-time PCR, loop-mediated isothermal amplification (LAMP), nucleic acid sequence-based amplification (NASBA) and others can potentially fill this gap by providing a sensitive and accurate identification of multiple *Plasmodium* species [10, 13]. DNA-based malaria assays in general, and PCR in particular, are significantly more sensitive than microscopy and RDTs. Limit of detection (LOD) for PCR is typically 1–5 parasites/µL [13–17] compared to 50–500 parasites/µL for microscopy [13, 14, 18] and more than 100 parasites/µL for RDT’s [14, 19]. Depending on the assay, PCR can be used for accurate species identification, detection of mixed infections, and parasite density estimation [20].

A novel, room temperature-stable, multiplex real-time PCR assay was used in this study to test blood samples from febrile patients in Bo, Sierra Leone. The Malaria Multiplex Sample Ready (MMSR) PCR assay can identify *P. falciparum* and *P. vivax*, and also detect presence of less common *Plasmodium* species via a universal malaria gene target. The results of this assay were compared with a well-characterized RDT assay and locally performed microscopy. The obtained results were consistent with previous observations that PCR-based tests have a significantly higher sensitivity when compared with both microscopy and RDTs [14, 16, 17]. In addition, the assay identified several malaria-positive samples that were negative for both *P. falciparum* and *P. vivax* malaria species. Further analysis of these *P. falciparum*/*P. vivax*-negative samples revealed the presence of other *Plasmodium* species (*P. ovale* and *P. malariae*) circulating within the tested population.

## Methods

### Subject recruitment and specimen collection

All patients seeking treatment at Mercy Hospital in Bo, Sierra Leone between February 5 and November 4, 2018, who had a clinically confirmed or self-reported fever starting not earlier than 10 days before the date of the interview, were invited to participate in the study, which was part of a bigger study testing the performance of experimental diagnostic devices. The research protocol used in this study was approved by the Sierra Leone Ethics and Scientific Review Committee and the institutional review boards of the US Naval Research Laboratory (NRL.2012.0007) and George Mason University (477605). Informed consent from patients (or, for children, consent from their parents) was obtained and documented prior to collection of clinical data or biological specimens. A total of 534 subjects consented to participate in the study. Samples of venous blood were collected from all study participants. In addition each subject participated in a survey which collected basic demographic information and also information related to previous infections, potential exposures and use of antibiotics before the enrolment. The participants were offered free standard-of-care testing at Mercy Hospital and referred for appropriate treatment when needed.

### Detection of malaria

For PCR-based detection, DNA extracted from venous blood (QIAamp DNA Mini Kit, Qiagen, Germantown, MD, USA) was analysed using MMSR (BioGX, Birmingham, AL, USA), a TaqMan-based real-time PCR assay. Developed and validated by researchers at Walter Reed Army Institute of Research (WRAIR) [21, 22], MMSR is designed to detect four targets in a single assay: *P. falciparum*, *P. vivax*, *Plasmodium* spp. (universal malaria target) and RNaseP (sample extraction control).

In parallel, venous blood samples were tested using the Malaria Ag Pf/Pan RDT (SD Bioline, Gyeonggi-do, Republic of Korea) performed according to manufacturer’s instructions. Samples were also analysed by microscopy using Giemsa-stained thick smears according to current World Health Organization (WHO) standard operating procedures [23]. At least 100 high power fields (HPFs) were examined for parasites. No parasite density was recorded for this study—just positive or negative parasitaemia determination. The slides were read by one microscopist, a second microscopist examined the sample in case of an ambiguous result. Both RDT testing and microscopy was performed by lab trained hospital technicians.

### Confirmation of malaria species identification

Specimens negative for both *P. falciparum* and *P. vivax* in the MMSR assay but with evidence of amplification of the universal malaria marker were retrospectively tested using a malaria speciation PCR assay [24]. This real-time SYBR green-based PCR assay uses six separate PCR primer sets to test for the presence of 18S rRNA universal malaria target and five species-specific targets: *P. falciparum* (Pf *r364*), *P. vivax* (Pv *dhfr*)*, P. malariae* (Pm *dhfr*)*, P. ovale* (Po *dhfr*) and *P. knowlesi* (Pk *r140*). The obtained 18S rRNA amplicons were sequenced for confirmatory identification of the detected species. All DNA sequencing was performed by Eurofins Genomics (Louisville, KY, USA).

## Results

### Malaria detection

**Fig. 1:**
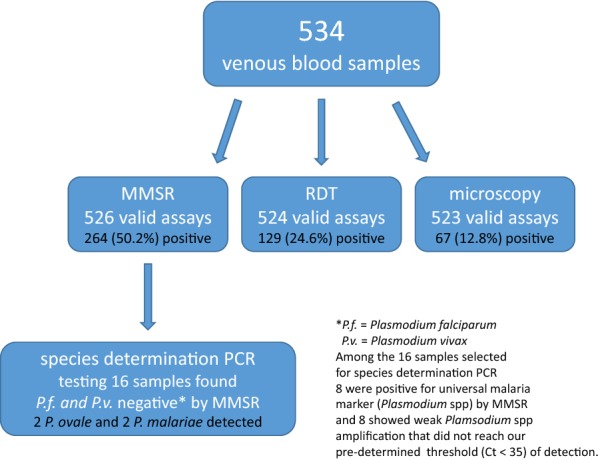
Numbers of samples tested by malaria diagnostic assays used in this study and numbers of valid results for each assay. The numbers and percentages of malaria positive samples are also shown

Table 1 and Fig. 1 summarize malaria detection results obtained by the three techniques used in this study. The MMSR assay produced valid results for 526 out of 534 tested specimens. At least one malaria marker was detected in approximately 50% of the samples with the vast majority of them positive for *P. falciparum,* while none were positive for *P. vivax*. The 87% concordance between *P. falciparum*-specific and universal malaria marker (*Plasmodium* spp.) detection was observed with the universal malaria marker identified in a noticeably smaller population of positive specimens (204 of 264, 77.3%). Only eight samples (1.5%) were positive for the universal malarial marker, but negative for both *P. falciparum* and *P. vivax*, suggesting the presence of other *Plasmodium* species.

**Table 1:** Results of malaria testing

Assay	Number of samples tested	Positive samples (percent of total)
MMSR		
Any *Plasmodium* marker	526	264 (50.2%)
*Plasmodium* spp.		204 (38.8%)
*P. falciparum*		256 (48.7%)
*P. vivax*		0 (0%)
SD bioline RDT		
Any *Plasmodium* marker	524	129 (24.6%)
*Plasmodium* spp.		29 (5.5%)
*P. falciparum*		129 (24.6%)
Microscopy (thick smears)	523	67 (12.8%)

As expected, the distribution of malaria-positive samples as detected by MMSR assay was not uniform among the tested subject age groups. A significantly larger percentage of children under age 15 were positive compared to other age groups (p < 0.001), Table 2.

**Table 2:** Results of malaria detection by MMSR. Data broken out by gender and age

Infection	Population	# Tested	# Positive	% Positive	p-value
Malaria (MMSR data)	All	526	264	50.2	–
	Male	206	106	51.4	0.667
	Female	319	158	49.5	
	Age 5–14	73	53	72.6	< 0.001
	Age 15–29	227	126	55.5	
	Age 30–44	117	46	39.3	
	Age 45+	105	37	36.2	

Among specimens tested by the commercial RDT, 524 of 534 (98.1%) produced valid results. Slightly less than one-fourth of them (129 samples, 24.6%) were positive for *P. falciparum*. Twenty-nine (22.5%) of these 129 were positive for both *P. falciparum* and *Plasmodium* spp. (universal malaria) antigens. No samples tested positive for *Plasmodium* spp. alone.

A total of 523 samples were analysed by light microscopy (Giemsa-stained thick blood smears). Among these samples, sixty-seven (12.8%) were found positive for *Plasmodium* spp. Species identification based on microscopic images of the parasites was not attempted.

Samples from 517 subjects were analysed with both MMSR and commercial RDT. Comparison of the results of *P. falciparum* detection showed a 71.2% concordance between the two methods. The low concordance could be mostly attributed to the lower apparent sensitivity of the RDT as compared to the MMSR PCR (Table 3). However, twelve samples (out of 128 RDT positive in this sample subset) were positive by RDT for *P. falciparum* but negative by MMSR.

**Table 3:** Concordance of SD Bioline RDT and MMSR for *P. falciparum* detection

	SD bioline RDT *P.f.* positive	SD bioline RDT *P.f.* negative	Total
MMSR *P.f.* positive	116	137	253
MMSR *P.f.* negative	12	252	264
Total	128	389	517

*P.f.Plasmodium falciparum*

**Fig. 2:**
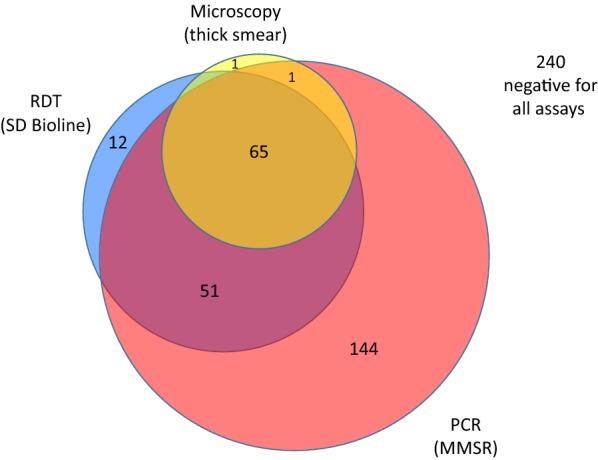
Comparison of MMSR, SD Bioline RDT and microscopy (smear) detection of malaria for samples with all three valid assays performed (n = 514). 240 samples tested negative using all the assays

A total of 514 samples were tested with all three techniques (MMSR, SD Bioline RDT and microscopy) with slightly more than 10% (65 of 514) positive by all three methods (Fig. 2). One sample found positive for malaria parasites by microscopy tested negative by both MMSR and RDT. Twelve samples (2.4%) tested positive by RDT only.

### Species identification in non-falciparum/non-vivax malaria

Among MMSR-tested specimens, eight were clearly positive for universal malaria marker but negative for both *P. falciparum* and *P. vivax*. An additional eight *P. falciparum*- and *P. vivax*-negative specimens showed evidence of universal malaria marker amplification, but did not reach the pre-determined threshold of detection (Ct < 35). All sixteen samples were retrospectively tested for the presence of other *Plasmodium* species using a real-time SYBR green-based PCR assay. The speciation PCR utilized six separate PCR amplifications to target markers specific for *P. falciparum*, *P. vivax*, *P. ovale*, *P. malariae* and *P. knowlesi* as well as a universal malaria marker (*Plasmodium* spp.). Positive results were obtained in nine of the sixteen samples tested. Amplicons with the expected melting temperatures indicated the presence of *P. falciparum* in five samples, *P. malariae* in two samples, and *P. ovale* in two samples. None of these nine samples were positive for more than one species of *Plasmodium*. DNA sequencing of the 16S (universal *Plasmodium*) amplicons detected in the same nine samples confirmed the *Plasmodium* species identification results.

## Discussion

This study explored the utility of a room temperature-stable, pre-aliquoted molecular assay for detection of malaria in a remote, low-resource location in West Africa. The MMSR PCR test was developed to detect multiple *Plasmodium* species and to identify the two predominant species responsible for human malaria, *P*. *falciparum* and *P. vivax* [1, 22]. The assay uses Sample-Ready™ format in which all necessary components (primers, probes, Taq polymerase and the reaction buffer) are provided as a freeze-dried pellet in a reaction tube compatible with most 0.1 mL real time PCR cycler blocks. The assay 8-tube strips are packed in pouches with desiccant allowing the tests to be stored at room temperature for months. DNA extraction needs to be performed prior to running the MMSR assay as is the case for the majority of PCR based methods. Depending on the method it usually requires several pipetting steps. On the other hand, preparation and use of MMSR assay, in contrast to many homebrew and commercial malaria PCR assays, requires only one pipetting—addition of 5 µL of DNA extracted from the clinical sample.

This study confirmed the earlier reports that approximately 50% of Mercy Hospital patients with a fever harbour *P. falciparum* parasites, as determined by PCR [25, 26]. *Plasmodium vivax* was not detected in any patients. The absence of *P. vivax* cases was expected, as this species is rarely encountered in West Africa [1]. However, two recent studies documented significant carriage of *P. vivax* DNA and/or antibodies against *P. vivax* proteins in some West African individuals; therefore, testing for this species has significant value [27, 28].

On the other hand, the presence of less common *P. ovale* and *P. malariae* was detected, each of these species accounting for at least 0.8% (2 of 264) of all malaria infections identified by the MMSR assay. The prevalence of *P. ovale* and *P. malariae* parasites is likely to be higher since only the 16 samples positive (or nearly positive) for the universal malaria, but negative for *P. falciparum* and *P. vivax* were tested, which excluded detection of any mixed infections in *P. falciparum*-positive samples. Mixed infections have been documented at low but significant numbers in West Africa [29–31]. This study demonstrates the possibility of a combined approach that detects and identifies all *Plasmodium* species, which may improve the effectiveness of treatment regimens (*P. vivax*, *P. ovale*) and lessen the burden of long-term morbidity (*P. malariae*) [1, 32].

The MMSR assay detected much greater numbers of malaria-positive samples than commercial RDT and microscopy. These results were consistent with earlier studies and the current knowledge on diagnostic sensitivity these techniques [14, 16, 17]; Kamau and colleagues [21] have determined limits of detection (LODs) of MMSR to be < 0.5 parasites/μL—significantly lower than LODs typically cited for RDTs and microscopy [16, 33, 34].

One of the analysed specimens was positive by microscopy only. It is unclear whether this microscopic observation was a false positive related to difficulties with slide preparation and examination or was due to the presence of other (non-malarial) parasites [35]. The accuracy and sensitivity of microscopy is known to rely on the quality of the smears (both thin and thick), the expertise of the microscopist, and the availability of high-quality, well-maintained light microscopes. Due to the inherent variability of these components, especially in resource-limited locations, RDTs are recommended instead of microscopy [36, 37]. This study reinforces that policy as RDTs detected malaria in approximately twice as many samples as microscopy.

A small number of samples that were malaria-positive by RDT were found negative by both microscopy and MMSR (n = 12). These samples may represent true malaria positives for which both MMSR and microscopy failed to detect the malaria parasites or they may be RDT false-positives. The presence of MMSR false-negatives is possible as a result of sequence variability in the PCR primer binding sites used by MMSR assay. The observation of five samples (distinct from these 12 RDT-positive/MMSR-negative samples) that were positive for *P. falciparum* by the PCR used for species identification but negative by MMSR shows that a small proportion of *P. falciparum* parasites in the tested population may evade detection by MMSR. An alternative explanation may be that the RDT detected residual malarial antigens that can circulate within the bloodstream for several weeks following clearing of the infection [38]. Additionally, non-specific (false-positive) results in malaria RDTs has been documented in subjects with underlying health conditions and other infectious diseases [39–41].

The MMSR assay used in this study is only one of many NAATs developed specifically for malaria detection but has several unique advantages. While costly (14.50 USD each), it offers wide coverage (detection of all and identification of two most prevalent *Plasmodium* species in a single reaction) and capability of measurement of the parasite density with room temperature stability and simple setup requiring just one pipetting [21, 22]. Most homebrew and commercially available PCR-based assays with comparable features (e.g. artus Malaria RG PCR by Qiagene or RealStar^®^ Malaria PCR Kits by Altona diagnostics) require transport and storage at − 20 °C and involve complex multi-step assay preparation. LAMP, a recently developed isothermal DNA amplification technology, holds promise of exceeding sensitivity of PCR-based malaria assays without the need for expensive and time consuming thermal cycling [42]. At least two different CE-marked commercial pan-malaria LAMP kits are available that are capable of detecting multiple *Plasmodium* species in ~ 60 min and require minimal hands-on manipulations for sample prep [43–45]. However, these pan-malaria LAMP kits—at their current stage of development—lack multiplexing capability (for species identification), have relatively high false positive rates compared to PCR, are still expensive on a per-sample basis, and have cold chain requirements [46, 47]. While sample prep for the MMSR is not as user-friendly as the commercial LAMP tests (with their associated sample prep kits), the MMSR costs less than $15 per reaction; this is significantly costlier than RDTs and some simpler DNA based assays (several US dollars per test) but less expensive than highly multiplexed, automated tests such as BioFire’s FilmArray diagnostic panels (> $100 per assay).

The high percentage of febrile patients at Mercy Hospital PCR positive for malaria in this and previous studies—combined with seasonal increases—suggest that a much larger part of the patient population is infected with *Plasmodium* than can be assumed based only on microscopy and RDT testing results [25, 26]. Application of PCR may allow us to identify a subpopulation with low density infections and milder malaria symptoms, who may not to be identified as malaria patients or receive anti-malarial treatment. Some researchers recently suggested that even submicroscopic and subclinical infections have significant negative impact on health and that all *Plasmodium*-positive persons should be treated [48]. However, the value of mass drug administration campaigns targeting all febrile cases or entire populations is disputed due to limited effectiveness, increased risk of adverse reactions to anti-malarials, costliness, and accelerated evolution of anti-malarial drug resistance [6, 49].

## Conclusions

Due to their cost and relative complexity, the WHO and malaria diagnostic community in general understandably do not consider NAATs—including PCRs—a viable alternative for routine diagnostics in most countries [50]. However, while experts are debating the clinical utility of detecting subclinical malaria infections [20, 51, 52], detection of low density parasitaemia in asymptomatic patients by DNA-based diagnostics offers significant epidemiological value, particularly if coupled with strategic elimination efforts; detecting subclinical infections allows more targeted and effective interventions [53]. Furthermore, tracking of the emergence and spread of anti-malarial drug resistance mutations and strains carrying these determinants can only be performed using the specificity inherent within nucleic acid-based technology [50, 54].

Although not yet approved for diagnostic use, the MMSR—and indeed any sensitive molecular test capable of detecting multiple *Plasmodium* species—represents a valuable tool for detection and epidemiological surveillance of malaria especially in low-resource areas directly affected and most impacted by this disease.

## Data Availability

The datasets used and/or analysed during the current study are available from the corresponding author on reasonable request.

## Abbreviations

LAMP: Loop-mediated isothermal amplification
MMSR: Multiplex Malaria Sample Ready assay
NAAT: Nucleic acid amplification test
NASBA: Nucleic acid sequence-based amplification
PCR: Polymerase chain reaction
RDT: Rapid diagnostic test
WHO: World Health Organization
WRAIR: Walter Reed Army Institute of Research
